# Supplementary figures and images for: Remodeling of the maternal gut microbiome during pregnancy is shaped by parity
Source: Microbiome. 2021 Jun 27;9:146. doi: 10.1186/s40168-021-01089-8 (PMC8237508; doi:10.1186/s40168-021-01089-8)

# SFig 1

A

Maturity Index Days 37-72

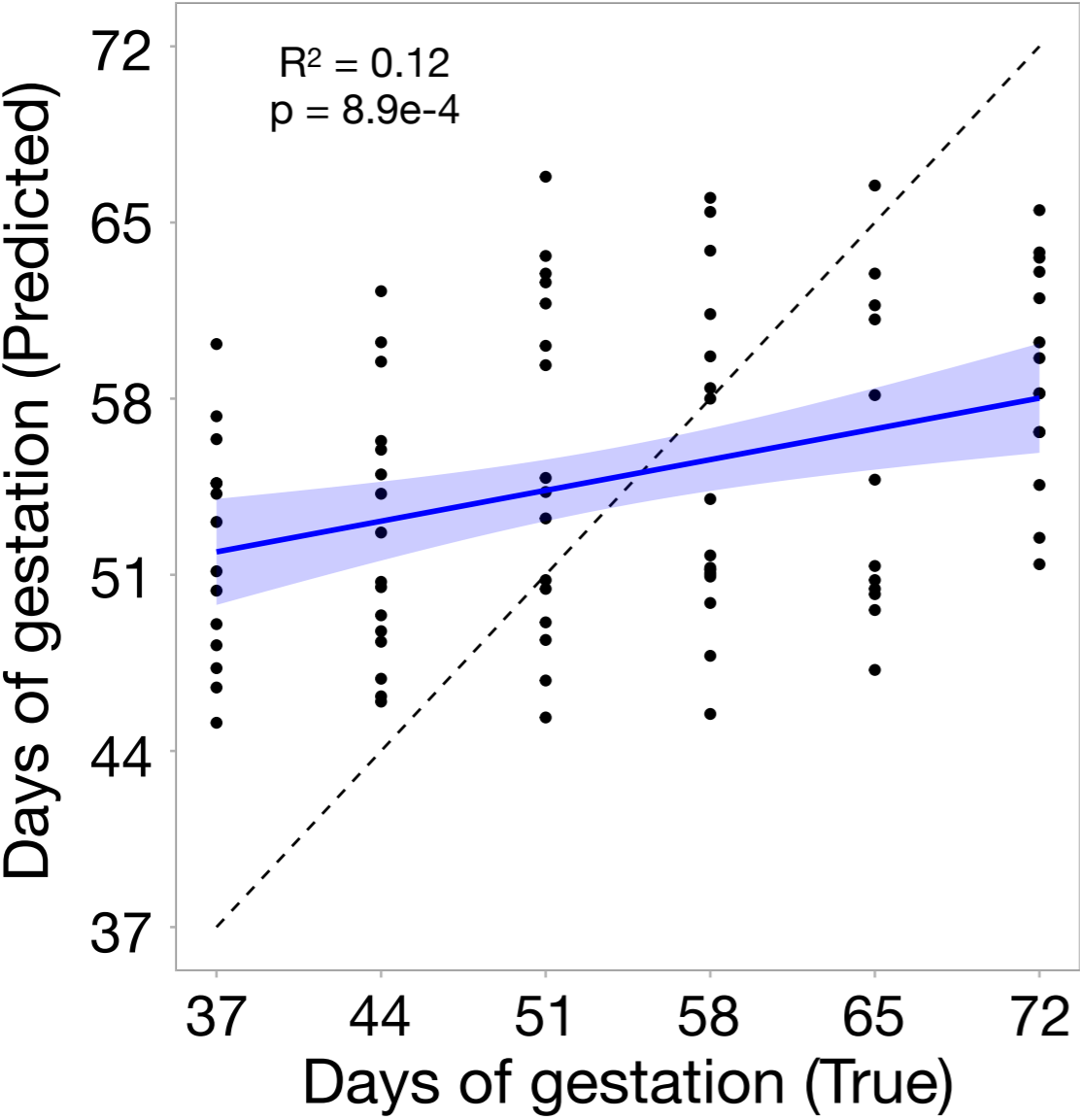

B

Maturity Index Days 79-114

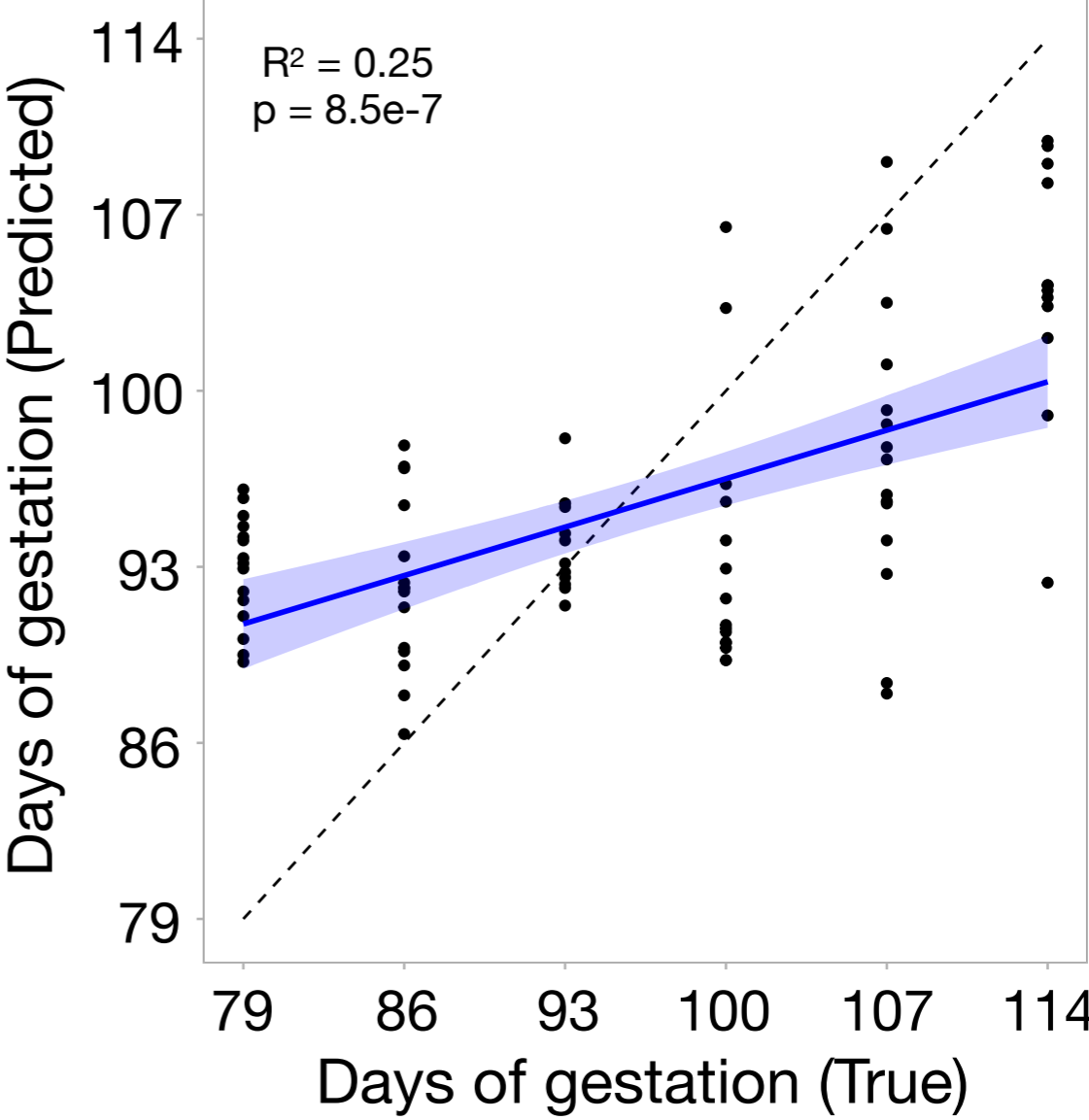

Supplement: Supplementary file 2 — Additional file 1: Supplemental Figure 1. Gut microbiota compositional changes more predictably after day 72 of pregnancy. A maturity index trained on 60% of the animals and tested on the remaining 40% from A) days 37 to 72 shows that the amount of time of gestation can be predicted (P = 8.9e-4) but with low accuracy (R2=0.12) and from B) days 79-114 shows that the amount of time of gestation can be predicted (P = 8.5e-7) with higher accuracy (R2=0.25). [file 40168_2021_1089_MOESM2_ESM.pdf]

# SFig 2

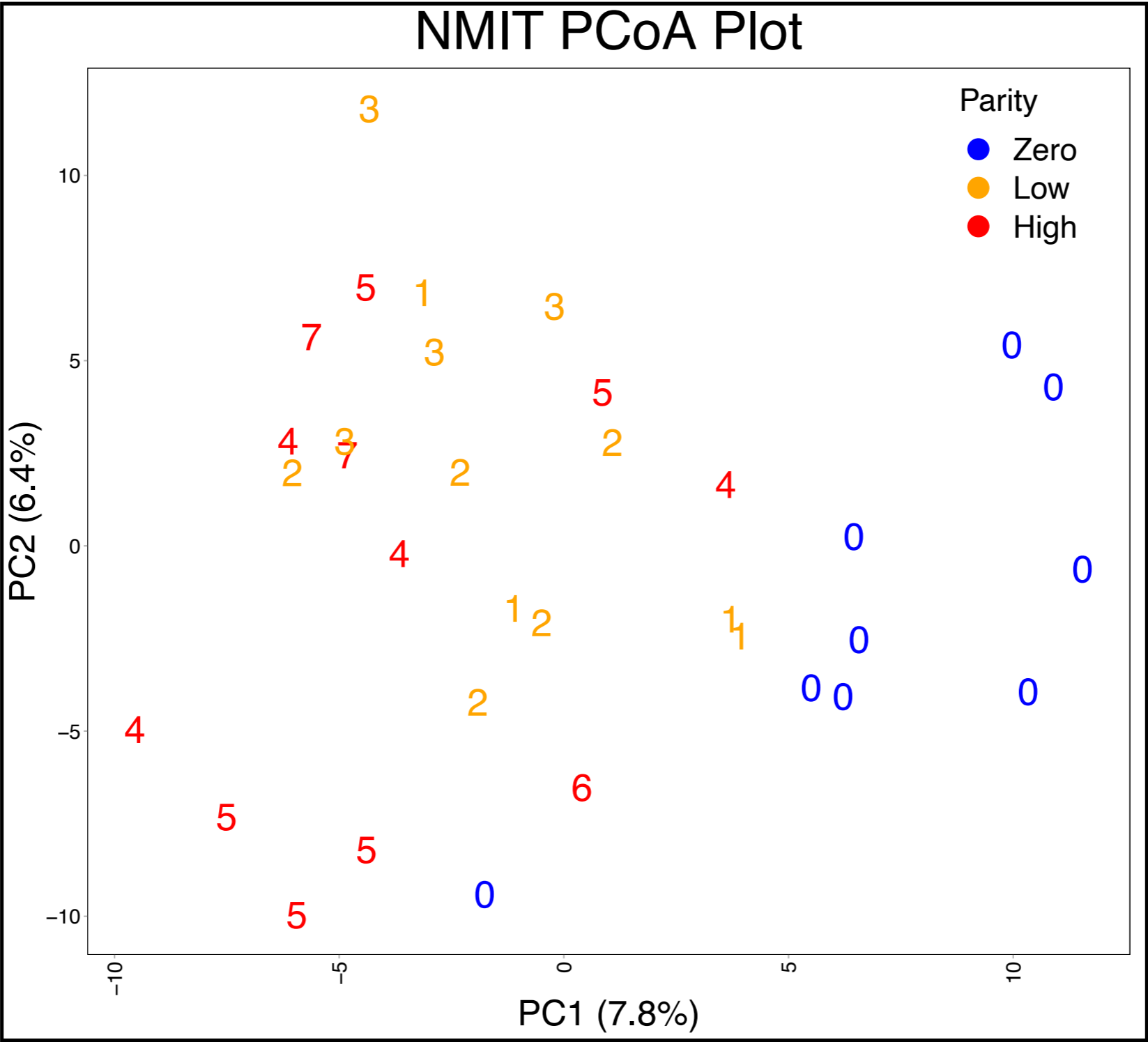

Supplement: Supplementary file 3 — Additional file 2: Supplemental Figure 2. The most significant difference between gut microbiota trajectories lie between nulliparous and multiparous animals. A Principal Coordinate Analysis (PCoA) plot of the NMIT data shows how the gut microbiota trajectory differs between animals of different parities across the first two axes. Each point represents an individual’s trajectory during gestation. Each point is a number which represents the parity, and the color of each number represents the parity bin (zero, low, or high). [file 40168_2021_1089_MOESM3_ESM.pdf]

# Shannon Alpha Diversity Over Time

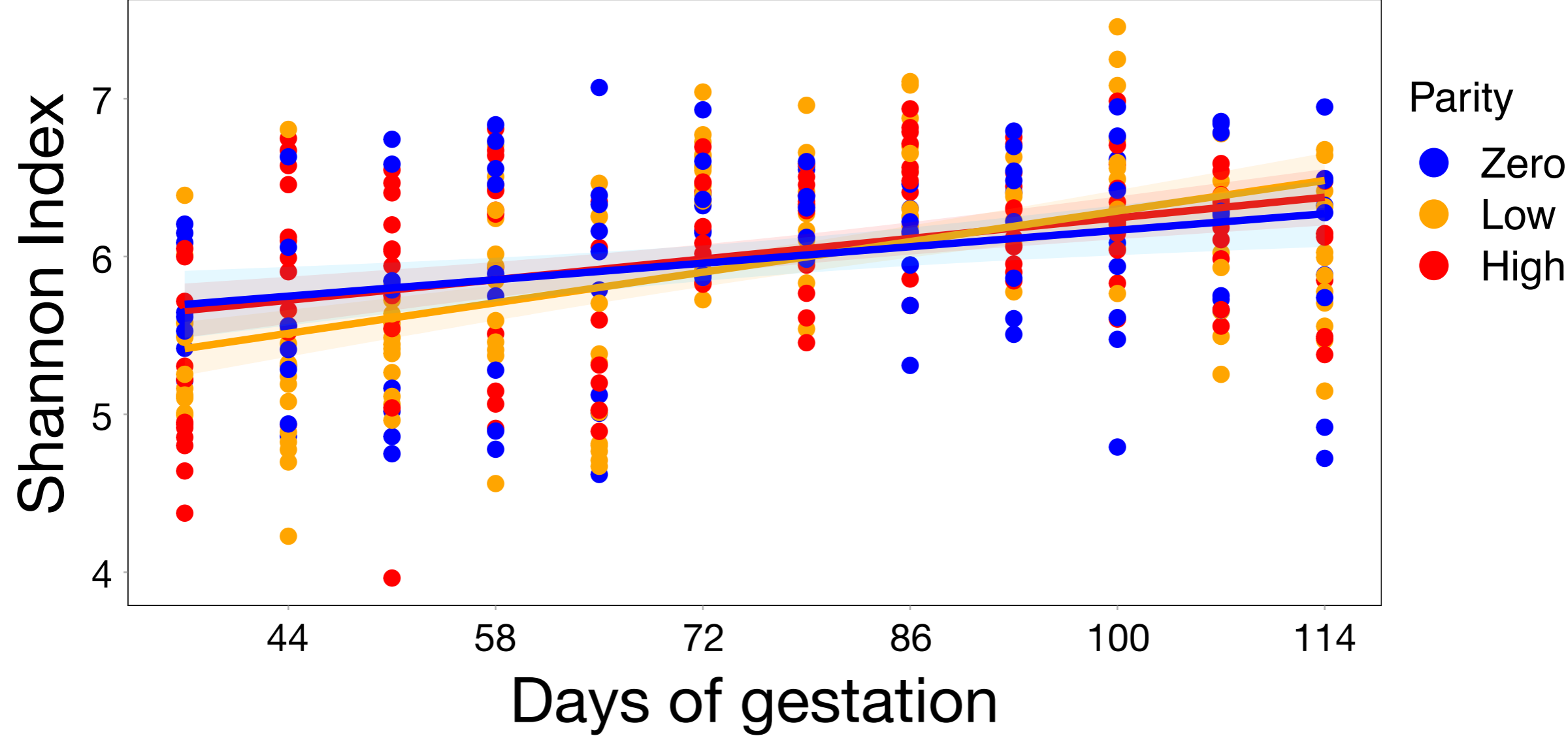

Supplement: Supplementary file 4 — Additional file 3: Supplemental Figure 3. Shannon alpha diversity increases throughout pregnancy across all parity groups. Shannon alpha diversity was calculated for each sample at each time point. Alpha diversity trends upwards over the course of pregnancy in all three parity groups. Parity does not associate with alpha diversity. [file 40168_2021_1089_MOESM4_ESM.pdf]

# SFig 4

A

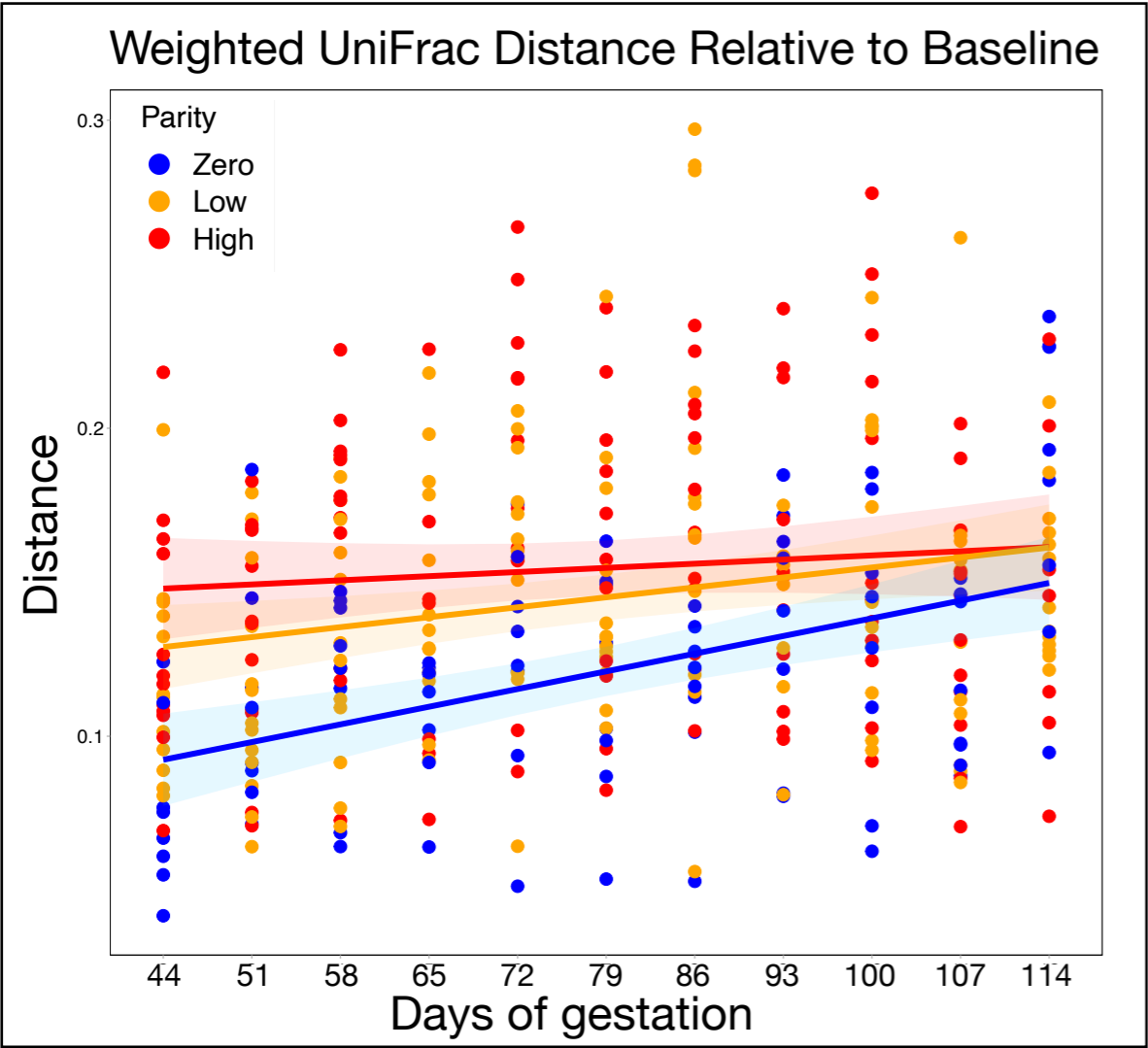

B

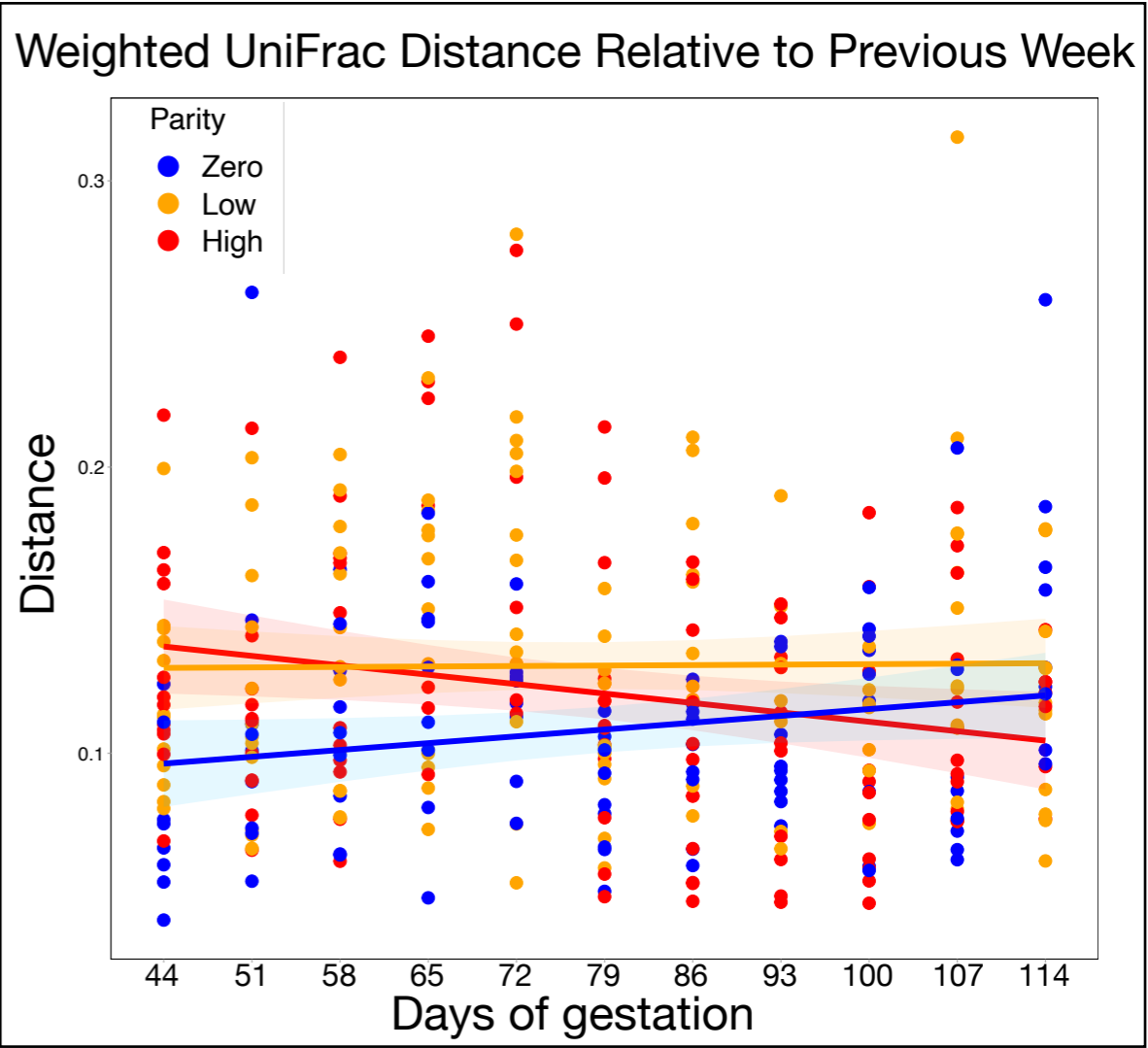

Supplement: Supplementary file 5 — Additional file 4: Supplemental Figure 4. Weighted UniFrac beta diversity shows that parity affects the gut microbiota trajectory during gestation. A) Weighted UniFrac beta diversity was calculated between each sample and the Day 37 sample from the same individual. B) Weighted UniFrac beta diversity was calculated between each sample and the previous week’s sample from the same individual. [file 40168_2021_1089_MOESM5_ESM.pdf]

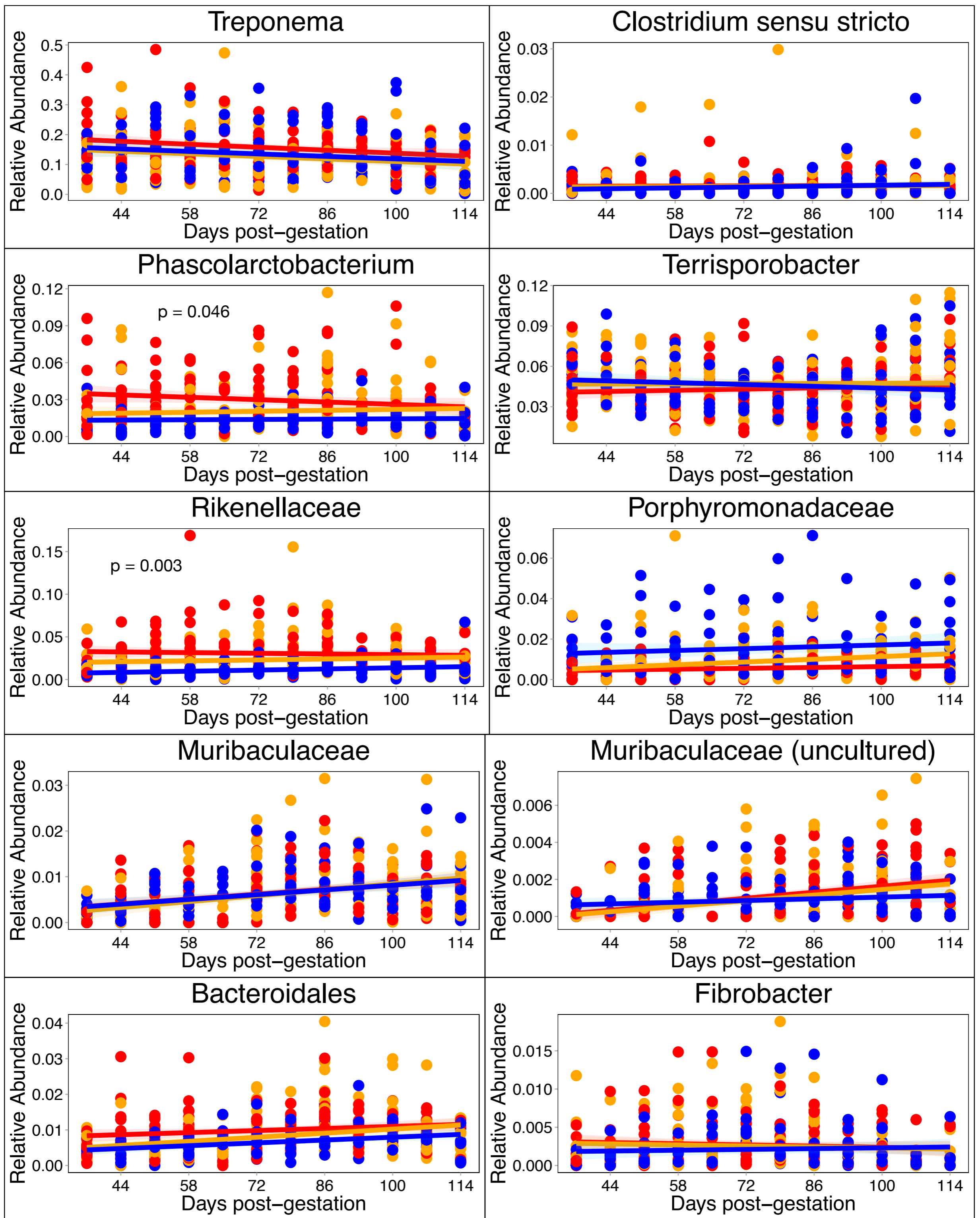

Supplement: Supplementary file 6 — Additional file 5: Supplemental Figure 5. The relative abundance of key bacterial taxa changes throughout gestation. A spline-fitting model was performed for the top five most important taxa from the DMM (Fig 1D) and the top five most important taxa from the maturity index (Fig 1B). The relative abundance of all ten taxa changed significantly during gestation (P < 0.01). Throughout gestation, the relative abundance of two taxa, the genus Phascolarctobacterium and the family Rikenellaceae, were significantly associated with parity (P < 0.05). [file 40168_2021_1089_MOESM6_ESM.pdf]

# SFig 6 A Sow vs. Piglet

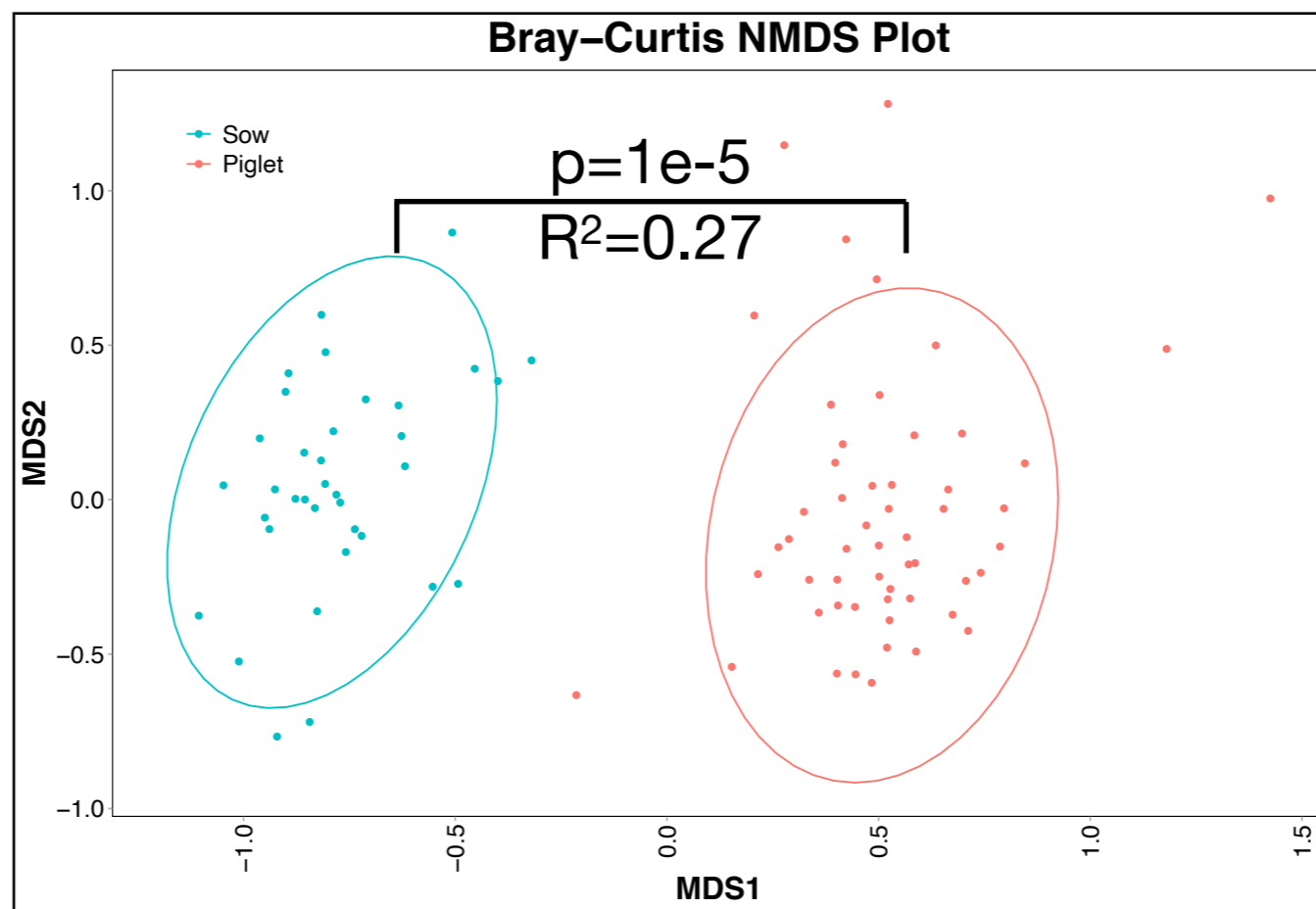

B

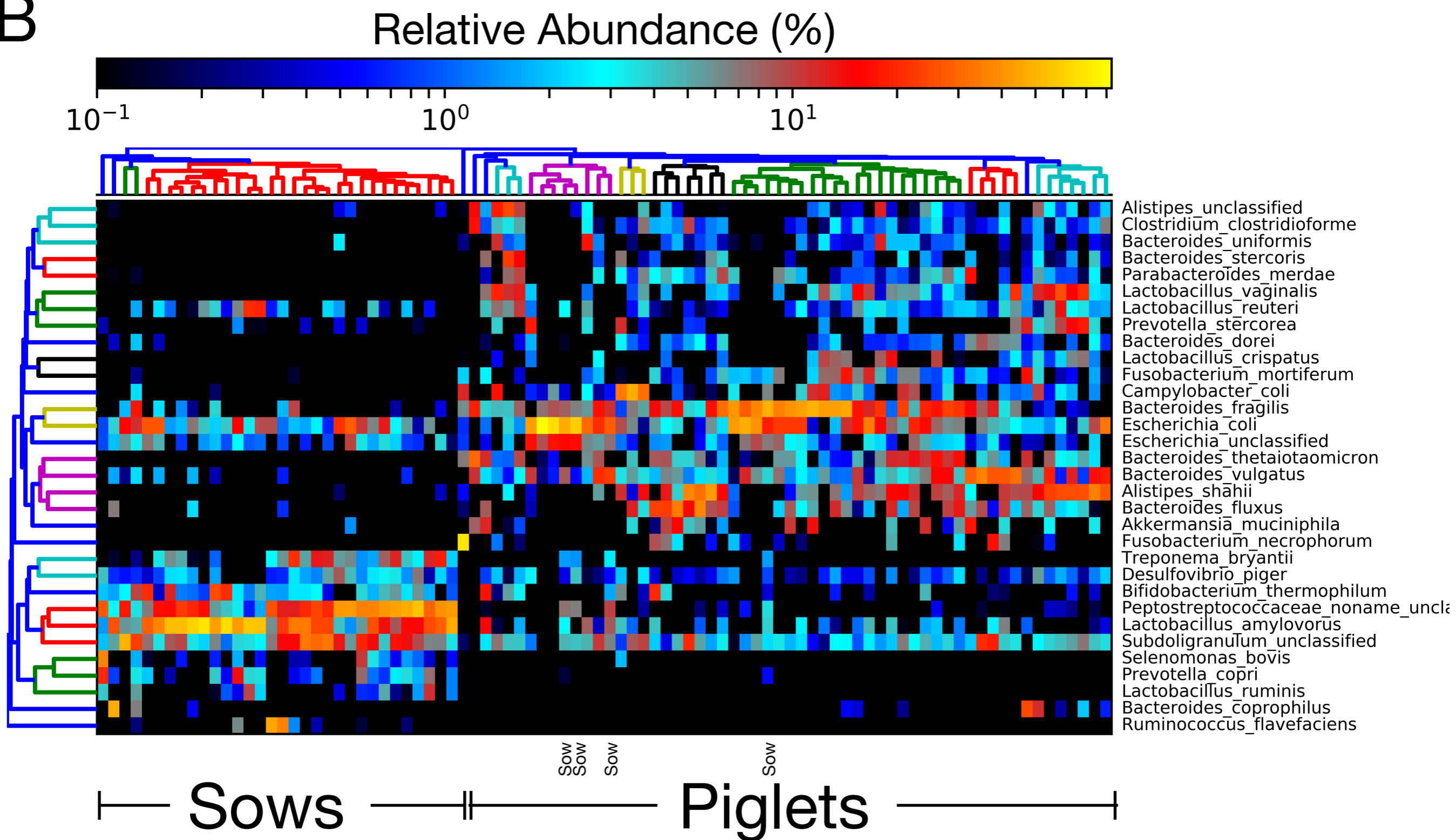

Supplement: Supplementary file 7 — Additional file 6: Supplemental Figure 6. Sows and piglets have significantly different gut microbiome compositions. Shotgun metagenomic sequencing was performed for fecal samples from 18 mother sows that were collected at days 37 and 114 of gestation, and from fecal swabs from 3 offspring of each pig collected 10 days after delivery. A) An NMDS plot representing the Bray-Curtis beta diversity between the 90 samples (36 sow and 54 piglet) was generated. The microbiome compositions of sows are significantly different from that of piglets (P = 1e-5). B) A heatmap was generated using the 32 bacterial species with relative abundance >1% among either sows or piglets. For each sample, the heatmap shows the relative abundance of each species. Consistent with the NMDS plot, the heatmap separates into two major phylogenetic branches, with sows on the left and piglets on the right. Four sow samples cluster within the piglet branch and are noted below the heatmap. [file 40168_2021_1089_MOESM7_ESM.pdf]

# SFig 8

## Phylogeny of E. coli from the sow and piglet gut

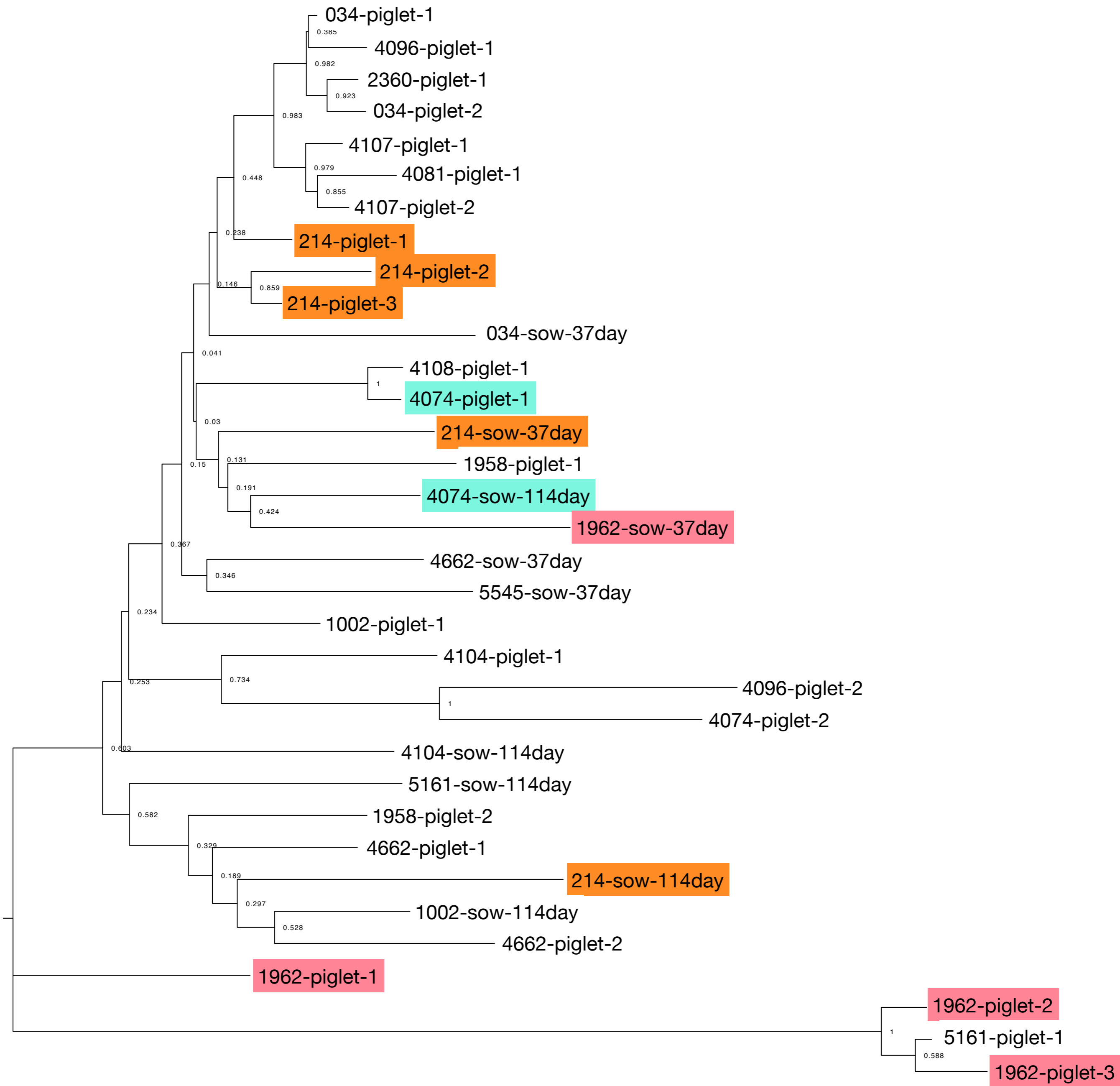

Supplement: Supplementary file 9 — Additional file 8: Supplemental Figure 8. There is no evidence that Escherichia coli in piglets were inherited from the maternal gut. A neighbor-joining phylogeny was reconstructed using 34 E. coli strains extracted from the shotgun metagenomic sequencing data from piglets and sows likely harboring only a single E. coli strain. Three sets of E. coli from different piglet-sow sets are highlighted in orange, cyan, and red. For example, highlighted in orange, the 3 piglets born to Sow 214 share similar E. coli genotypes, but immediately prior to birth (Day 114), the sow’s gut harbored a very different E. coli genotype. Similarly, in cyan, Sow 4074 and her piglet harbor different E. coli genotypes. Finally, in red, three piglets of Sow 1962 harbor E. coli genotypes with a most recent common ancestor at the root of the phylogeny. E. coli was chosen because it is the microbe found in the highest abundance across both sows and piglets, thus providing the deepest coverage of strains. [file 40168_2021_1089_MOESM9_ESM.pdf]

SFig 9

A

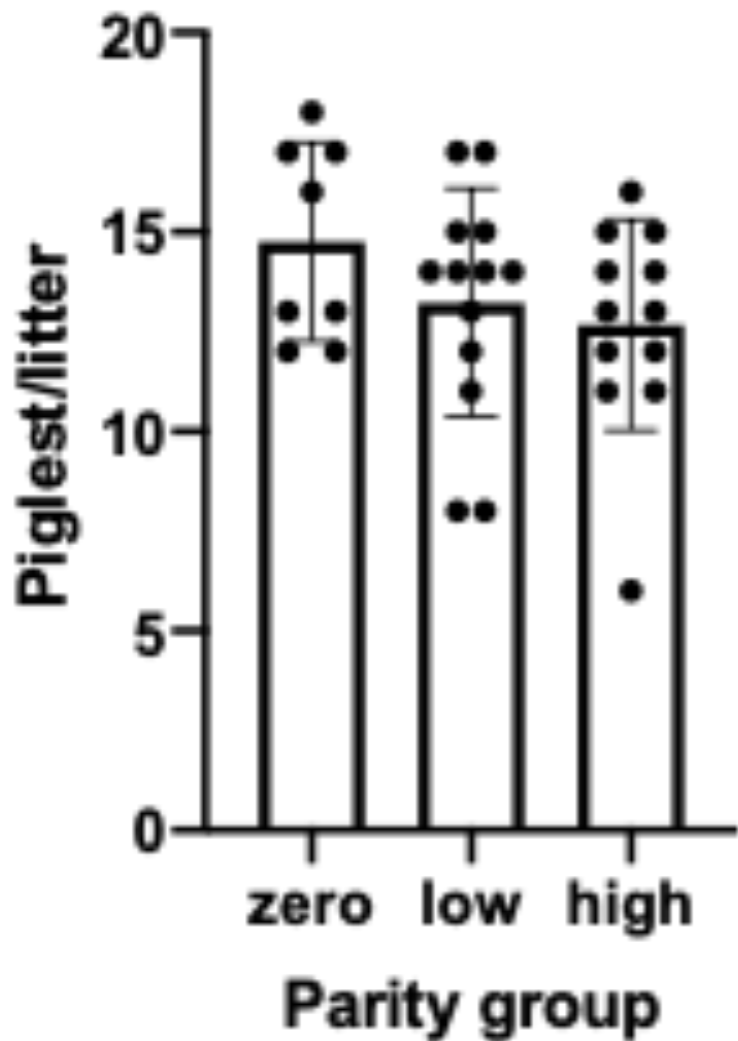

B

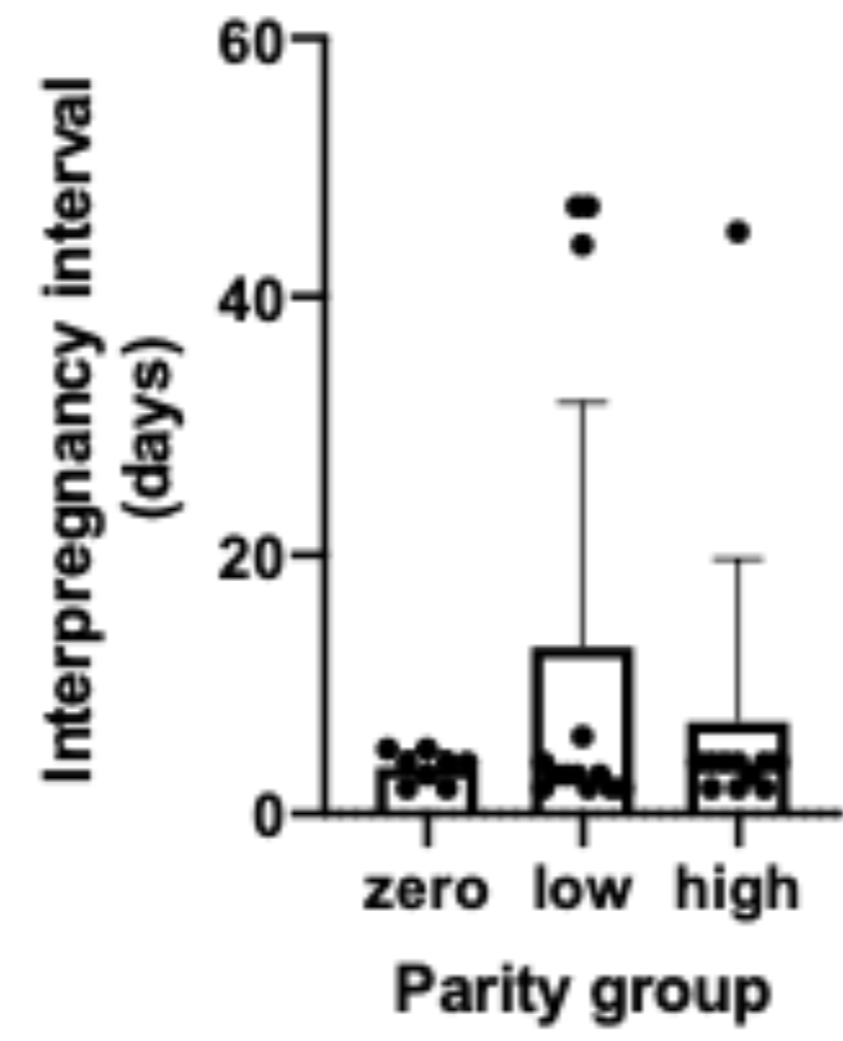

C

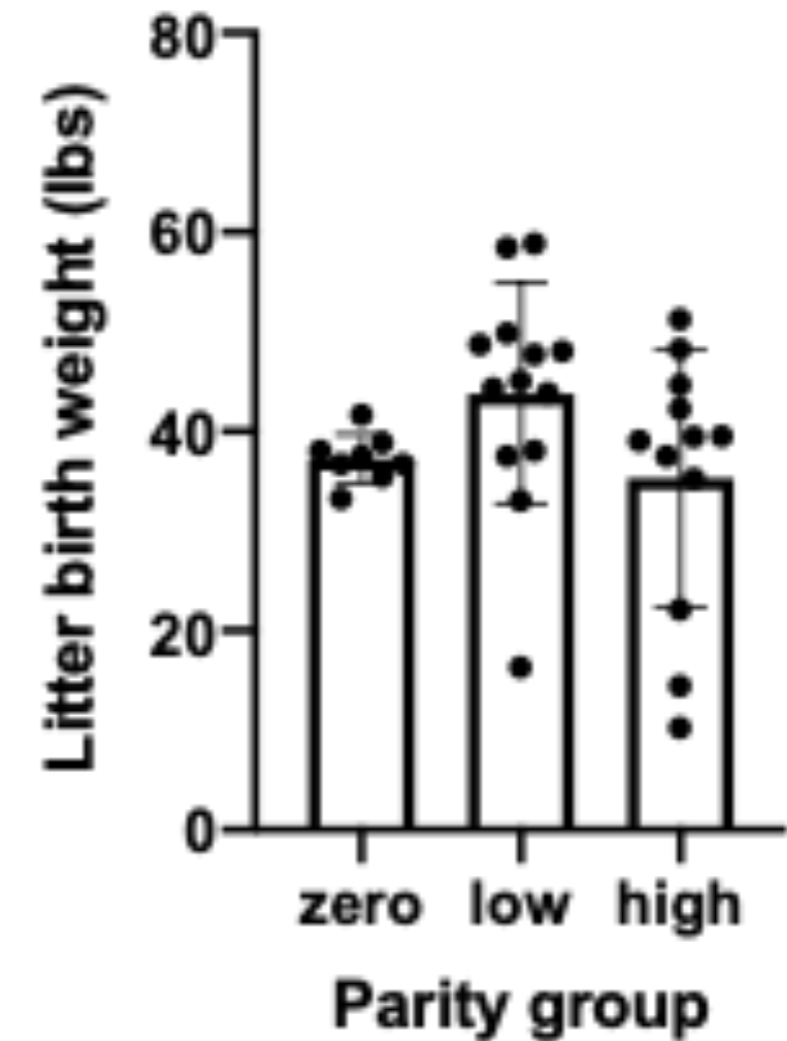

Supplement: Supplementary file 10 — Additional file 9: Supplemental Figure 9. The number of piglets per litter, the interpregnancy interval, and litter weight are not significantly different between parity groups. A) The number of piglets per litter across parity groups is constant. B) The number of days between weaning and insemination does not differ between parity groups. C) The combined weight of each litter is not significantly different between parity groups. All P-values are > 0.05. [file 40168_2021_1089_MOESM10_ESM.pdf]
