# Supplementary material for: Remodeling of the maternal gut microbiome during pregnancy is shaped by parity
Source: Microbiome. 2021 Jun 27;9:146. doi: 10.1186/s40168-021-01089-8 (PMC8237508; doi:10.1186/s40168-021-01089-8)
Supplement: Supplementary file 8 — Additional file 7: Supplemental Figure 7. The relative abundance of bacterial taxa in a sow does not correlate with the relative abundance of that taxa in her offspring. Linear regression analyses was performed for each of the 8 bacterial species with average relative abundance >1% across all samples and that were present in at least two-thirds of all samples. For each piglet, the relative abundance of each bacterial species in its mother at Day 114 was plotted (X-axis) against the relative abundance of that species in the piglet 10 days after delivery (Y-axis). Fecal swabs from three piglets from each sow were sequenced. There were no significant correlations between bacterial species in mother and offspring in any of the eight species (Adj. P > 0.3). [file 40168_2021_1089_MOESM8_ESM.pdf]

SFig 7

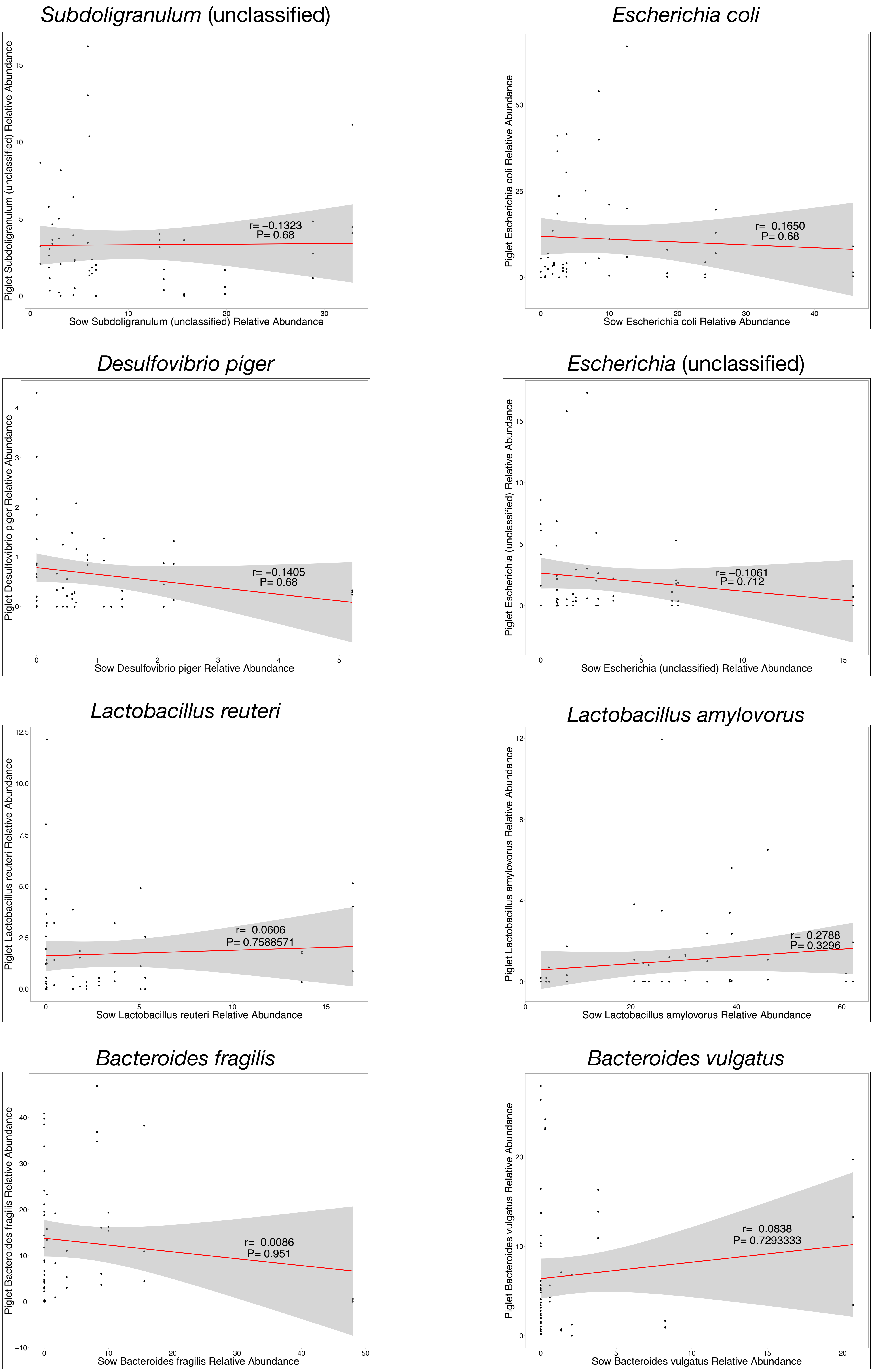
Subdoligranulum (unclassified)
Piglet Subdoligranulum (unclassified) Relative Abundance
Sow Subdoligranulum (unclassified) Relative Abundance
r= −0.1323
P= 0.68
Escherichia coli
Piglet Escherichia coli Relative Abundance
Sow Escherichia coli Relative Abundance
r= 0.1650
P= 0.68
Desulfovibrio piger
Piglet Desulfovibrio piger Relative Abundance
Sow Desulfovibrio piger Relative Abundance
r= −0.1405
P= 0.68
Escherichia (unclassified)
Piglet Escherichia (unclassified) Relative Abundance
Sow Escherichia (unclassified) Relative Abundance
r= −0.1061
P= 0.712
Lactobacillus reuteri
Piglet Lactobacillus reuteri Relative Abundance
Sow Lactobacillus reuteri Relative Abundance
r= 0.0606
P= 0.7588571
Lactobacillus amylovorus
Piglet Lactobacillus amylovorus Relative Abundance
Sow Lactobacillus amylovorus Relative Abundance
r= 0.2788
P= 0.3296
Bacteroides fragilis
Piglet Bacteroides fragilis Relative Abundance
Sow Bacteroides fragilis Relative Abundance
r= 0.0086
P= 0.951
Bacteroides vulgatus
Piglet Bacteroides vulgatus Relative Abundance
Sow Bacteroides vulgatus Relative Abundance
r= 0.0838
P= 0.7293333
